# Dural Plasmacytoma with Meningeal Myelomatosis in a Patient with Multiple Myeloma

**DOI:** 10.1155/2018/6730567

**Published:** 2018-02-13

**Authors:** Nieves Gascón, Héctor Pérez-Montero, Sandra Guardado, Rafael D'Ambrosi, María Ángeles Cabeza, José Fermín Pérez-Regadera

**Affiliations:** Radiation Oncology Department, Hospital Universitario 12 de Octubre, Madrid, Spain

## Abstract

Here, we describe the case of a 66-year-old male diagnosed with multiple myeloma who presented with generalized tonic-clonic seizures. Magnetic resonance imaging demonstrated a right solid extra-axial parieto-occipital lesion with typical characteristics of meningeal myelomatosis. Biopsy was performed, which diagnosed a dural plasmacytoma. Because of this, we started concomitant therapy with radiotherapy and lenalidomide, but the patient has a poor response to treatment and died few weeks after its initiation. Myelomatous involvement of the dura mater is a rare occurrence, given that only few cases were reported in the English literature. This presentation confers an ominous prognosis and must be a suspect diagnosis in patients diagnosed with multiple myeloma presenting neurological symptoms.

## 1. Introduction

Plasma cell tumors are characterized by proliferation of monoclonal plasma cells. They may appear as single lesions (solitary plasmacytoma) or multiple ones (multiple myeloma). Plasmacytomas usually develop in the bone, although they may also do so in soft tissues (called extramedullary plasmacytoma). Extramedullary plasmacytomas can arise without evidence of multiple myeloma or also in patients with multiple myeloma at any time during the course of the disease [1].

Extramedullary plasmacytoma appears most frequently in head and neck locations [2], although cases have also been reported in the upper aerodigestive tract, gastrointestinal tract, urinary bladder, central nervous system, thyroids, breast, testicles, parotid gland, lymph nodes, and skin. They represent approximately 3% of plasma cell tumors, with median age at diagnosis being 55–60. Around 2/3 of the patients are male [3, 4]. Clinical presentation depends on lesion location, direct involvement of structures or organs, or their compression [5]. Extramedullary plasmacytomas with an intracranial location can develop from the cranium, meninges, or parenchyma. Meningeal involvement is a rare presentation with an ominous prognosis for patients [6].

We present a rare case of a patient with multiple myeloma that developed a secondary cranial plasmacytoma with meningeal involvement in the form of myelomatosis.

## 2. Case Study

A 66-year-old male patient presented with a history of liver transplant in 1993 due to severe acute hepatic insufficiency. Six months after transplant, he developed acute rejection with Epstein–Barr virus (EBV) viremia, requiring aggressive immunosuppression with cyclosporine and corticosteroids.

In 2009, due to generalized musculoskeletal pain not responding to analgesics, he was diagnosed with stage IIA multiple myeloma. He initiated treatment with bortezomib-dexamethasone receiving 5 cycles, after which he achieved complete remission (with disappearance of monoclonal spike in blood and urine, negative electrophoresis, and negative immunofixation).

In January 2013, he presented with new-onset pain in the left hip, for which a pelvic MRI was conducted which showed a lesion in the left greater trochanter compatible with a multiple myeloma secondary lesion. Treatment with bortezomib-dexamethasone was resumed with very poor tolerance leading to severe hydroelectrolytic disorders, altered bowel function, and pneumonia. At that moment, treatment was interrupted for this reason and only monthly zoledronic acid was maintained.

In May 2014, he presented with reappearance of IgA monoclonal spike, and therefore treatment with bortezomib-dexamethasone was reinitiated with excellent tolerance and significant diminishment of pain in the left femur. He completed 4 cycles and then had bortezomib-dexamethasone every 2 months and concomitant zoledronic acid monthly.

**Figure 1:**
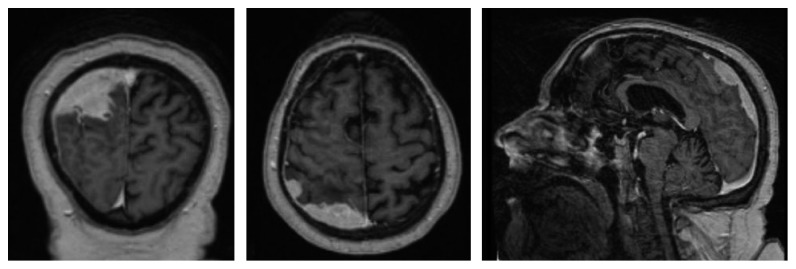
MRI showing right solid extra-axial parieto-occipital lesion with typical characteristics of meningeal myelomatosis. The tumor adapts to the underlying brain surface, is intensely enhanced with contrast, and shows adjacent pachymeningeal enhancement with focal spread through leptomeninges towards sulci in the proximal convexity, associated with vasogenic edema but not causing intracranial herniation.

**Figure 2:**
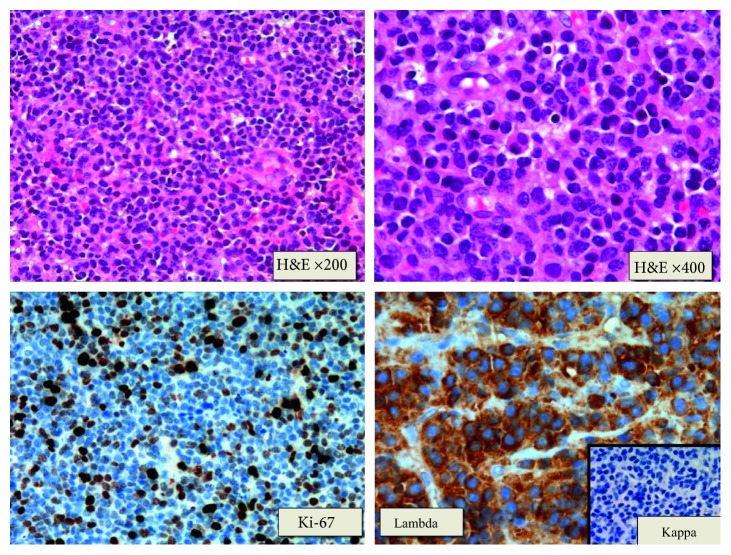
Pathological study: tumor proliferation of diffuse growth plasma cells made up of atypical plasma cells. The tumor immunophenotype shows lambda light chain restriction. The rest of the markers analyzed were negative (EMA, CD56, CD117, CD20, CD79a, and CD3). The Ki-67 proliferative index was approximately 30–40%.

On November 8, 2015, he was admitted to the Neurosurgery Unit due to generalized tonic-clonic seizures accompanied by sialorrhea, gaze deviation, and disorientation, with subsequent postcritical period and without sphincter relaxation. A cranial MRI was performed to investigate these symptoms. This study revealed a right solid extra-axial parieto-occipital lesion with typical characteristics of meningeal myelomatosis. The tumor adapted to the underlying brain surface showing adjacent pachymeningeal enhancement with focal spread through leptomeninges (Figure 1). A biopsy of the cranial lesion was conducted on November 12, 2015, with pathological diagnosis of plasmacytoma (Figure 2). Given the existence of meningeal myelomatosis, a spine MRI was requested that ruled out spread to this level.

Finally, it was decided to administer radiotherapy on the right parieto-occipital meningeal lesion, prescribing a total initial dose of 40 Gy with a fractionation of 2 Gy per day. Radiotherapy was administered concurrently with lenalidomide (15 mg a day) and dexamethasone (20 mg a week).

At the time 16 Gy had been received, the patient was hospitalized due to progressive deterioration of his general condition together with cognitive impairment, diminished mobility, urinary incontinence, and grade 4 thrombocytopenia. During admission, the patient deteriorated progressively with the increase of these symptoms and the appearance of clinical signs of intracranial hypertension. Given this situation and in agreement with the Hematology Service, we decided to interrupt active treatment and request evaluation from the Palliative Care Service.

## 3. Discussion

There are very few cases described in literature of intracranial involvement of plasmacytoma and multiple myeloma. Dural involvement without spreading from the bone and meningeal spreading of the disease are even rarer scenarios [1, 7, 8]. There is theory that this meningeal involvement may be present from the start of the disease, increasing its presence during development of this pathology. This is based on the fact that most treatments for myeloma do not cross the blood-brain barrier [9–11].

Our case is a patient diagnosed with multiple myeloma who subsequently developed a secondary dural plasmacytoma; this is usually a benign scenario with relative good prognosis. Nevertheless, this case further developed meningeal myelomatosis, which changed the course of the disease giving it an ominous prognosis.

The main diagnosis of dural plasmacytoma should be based on clinical suspicion, which shall depend on the region where the lesion is located and its extension. Clinical presentation of plasmacytomas in the dura mater is usually related to space occupation such as headache, seizures, or focal neurological deficit [1, 5, 7, 12–15]. Diagnosis should be conducted with imaging tests: in a CT scan, it is possible to confirm signal iso- or hyperintensity. An MRI shows T1 signal iso- or hyperintensity and marked T2 signal hypointensity. This presentation mimics the image of a meningioma, which is the main differential diagnosis together with metastases and lymphoma [10–12, 15]. Therefore, a histological study is essential to confirm the diagnosis. Tumor proliferation of diffuse growth atypical plasma cells with lambda chain positivity is characteristic [1, 15].

Apart from extramedullary plasmacytoma, our patient had underlying liver transplantation and long-standing immunosuppression. An increased risk of transplantation-related malignancies has been described in patients in this situation [16–19], the most common being nonmelanoma skin cancers and non-Hodgkin's lymphoma. Despite its rarity, transplant patients carry a higher risk of multiple myeloma as well. Transplant-associated non-Hodgkin's lymphomas are also commonly associated with EBV infection in extranodal sites [16]. In addition, an association between cyclosporine treatment and the development of malignancy is widely known [16, 17]. Otherwise, prognosis of transplant-related neoplasms in comparison to standard malignancies has not been well studied [18, 19].

Due to its rarity, the definitive treatment for these patients is not well known. Given its high radiosensitivity, reasonable options include radiotherapy given with curative intent, or surgical resection of the plasmacytoma, as thorough as possible, followed by adjuvant radiotherapy. The optimal doses of radiotherapy are controversial due to the scarcity of publications, but authors seem to agree that, in the absence of surgery, the minimum advisable is a total dose of 40–50 Gy given with conventional fractionation [1, 12–15].

In our case, the surgical option was discarded due to the extensive dural affectation and to the medical history of the patient, fundamentally the state of immunosuppression presented. As this was ruled out, we had to explore other therapeutic options, and together with Hematology Service, we decided to provide combined treatment with radiotherapy and lenalidomide. We based this decision on current literature supporting this drug to manage intracranial involvement of these lesions [20]. We decided to prescribe 40 Gy due to the fragile status of the patient and the use of concomitant lenalidomide. Unfortunately, during its administration, the patient presented with severe deterioration of his general condition and treatment had to be interrupted. As in previous literature, describing median survival of 6 weeks [8, 21], the meningeal involvement gave the patient an ill-fated prognosis in spite of the local and systemic treatments.

This ominous outcome in the short term may be due mainly to the massive dural involvement that affected a large part of the central nervous system. Additionally, the patient had undergone a transplant with its associated complications and had suffered a long illness receiving numerous lines of chemotherapy. This background had deteriorated its baseline situation, preventing surgical treatment and decreasing tolerance to the aggressive treatments carried out in this case. This poor tolerance caused severe clinical deterioration during admission showing intracranial hypertension symptoms and a worsening of meningeal symptoms. This deterioration precluded further active treatment.

Although dural involvement of a multiple myeloma is a very infrequent situation, it should be suspected in patients with this disease that present neurological symptoms. It is essential to conduct an imaging test, preferably an MRI to reach the diagnosis. Treatment for these patients has not been clearly defined to date. Meningeal involvement gives it an ill-fated prognosis.

## References

[1] Cerase A., Tarantino A., Gozzetti A. (2008). Intracranial involvement in plasmacytomas and multiple myeloma: a pictorial essay. *Neuroradiology*.

[2] Creach K. M., Foote R. L., Neben-Wittich M. A., Kyle R. A. (2009). Radiotherapy for extramedullary plasmacytoma of the head and neck. *International Journal of Radiation Oncology∗Biology∗Physics*.

[3] Dores G. M., Landgren O., McGlynn K. A., Curtis R. E., Linet M. S., Devesa S. S. (2009). Plasmacytoma of bone, extramedullary plasmacytoma, and multiple myeloma: incidence and survival in the United States, 1992–2004. *British Journal of Haematology*.

[4] Frassica D. A., Frassica F. J., Schray M. F., Sim F. H., Kyle R. A. (1989). Solitary plasmacytoma of bone: Mayo Clinic experience. *International Journal of Radiation Oncology∗Biology∗Physics*.

[5] Kilciksiz S., Karakoyun-Celik O., Agaoglu F. Y., Haydaroglu A. (2012). A review for solitary plasmacytoma of bone and extramedullary plasmacytoma. *The Scientific World Journal*.

[6] Chamberlain M. C., Glantz M. (2008). Myelomatous meningitis. *Cancer*.

[7] Schwartz T. H., Rhiew R., Isaacson S. R., Orazi A., Bruce J. N. (2001). Association between intracranial plasmacytoma and multiple myeloma: clinicopathological outcome study. *Neurosurgery*.

[8] Bladé J., Rosiñol L. (2007). Complications of multiple myeloma. *Hematology/Oncology Clinics of North America*.

[9] Laribi K., Mellerio C., Baugier A. (2015). Meningeal involvement in multiple myeloma. *Clinical Case Reports*.

[10] Hirata K., Takahashi T., Tanaka K. (1996). Leptomeningeal myelomatosis in well-controlled multiple myeloma. *Leukemia*.

[11] Sham R. L., Phatak P. D., Kouides P. A., Janas J. A., Marder V. J. (1999). Hematologic neoplasia and the central nervous system. *American Journal of Hematology*.

[12] Manabe M., Kanashima H., Yoshii Y. (2010). Extramedullary plasmacytoma of the dura mimicking meningioma. *International Journal of Hematology*.

[13] Azarpira N., Noshadi P., Pakbaz S., Torabineghad S., Rakei M., Safai A. (2014). Dural plasmacytoma mimicking meningioma. *Turkish Neurosurgery*.

[14] Hasturk A. E., Basmaci M., Erten F., Cesur N., Yilmaz E. R., Kertmen H. (2013). Solitary dural plasmacytoma mimicking meningioma and invading calvarium. *Journal of Craniofacial Surgery*.

[15] Khalili R. P., Mokhtari M., Fard S. A., Neshat A., Norouzi R. (2015). Solitary dural plasmacytoma with parenchymal invasion. *Asian Journal of Neurosurgery*.

[16] Pacheco T. R., Hinther L., Fitzpatrick J. (2003). Extramedullary plasmacytoma in cardiac transplant recipients. *Journal of the American Academy of Dermatology*.

[17] Dempewolf R., Lee J. H. (2008). Extramedullary plasmacytoma presenting as a nasal mass in an immunosuppressed patient: treatment after failed primary radiotherapy. *Ear Nose and Throat Journal*.

[18] Wilberger A. C., Prayson R. A. (2015). Intracranial involvement of posttransplant lymphoproliferative disorder multiple myeloma. *Journal of Clinical Neuroscience*.

[19] Aoki T., Kasai M., Harada Y. (2013). Stable renal engraftment in a patient following successful tandem autologous/reduced-intensity conditioning allogeneic transplantation for treatment of multiple myeloma with del(17p) that developed as a post-transplantation lymphoproliferative disease following renal transplantation. *International Journal of Hematology*.

[20] Devoe C. E., Li J. Y., Demopoulos A. M. (2014). The successful treatment of a recurrent intracranial, dural-based plasmacytoma with lenalidomide. *Journal of Neuro-Oncology*.

[21] Petersen S. L., Wagner A., Gimsing P. (1999). Cerebral and meningeal multiple myeloma after autologous stem cell transplantation: a case report and review of the literature. *American Journal of Hematology*.

